# *MeNPF4.5* Improves Cassava Nitrogen Use Efficiency and Yield by Regulating Nitrogen Uptake and Allocation

**DOI:** 10.3389/fpls.2022.866855

**Published:** 2022-04-25

**Authors:** Qiongyue Liang, Mengmeng Dong, Minghua Gu, Peng Zhang, Qiuxiang Ma, Bing He

**Affiliations:** ^1^State Key Laboratory for Conservation and Utilization of Subtropical Agro-Bioresources, College of Agriculture, Guangxi University, Nanning, China; ^2^National Demonstration Center for Experimental Plant Science Education, College of Agriculture, Guangxi University, Nanning, China; ^3^National Key Laboratory of Plant Molecular Genetics, CAS Center for Excellence in Molecular Plant Sciences, Chinese Academy of Sciences, Shanghai, China

**Keywords:** cassava, nitrate transport, nitrogen efficiency, growth, *MeNRT1.1*/*MeNPF4.5*

## Abstract

Improving nitrogen use efficiency (NUE) is a very important goal of crop breeding throughout the world. Cassava is an important food and energy crop in tropical and subtropical regions, and it mainly use nitrate as an N source. To evaluate the effect of the nitrate transporter gene *MeNPF4.5* on the uptake and utilization of N in cassava, two *MeNPF4.5* overexpression lines (*MeNPF4.5* OE-22 and *MeNPF4.5* OE-34) and one *MeNPF4.5* RNA interference (RNAi) line (*MeNPF4.5* Ri-1) were used for a tissue culture experiment, combining with a field trial. The results indicated that MeNPF4.5 is a plasma membrane transporter mainly expressed in roots. The gene is induced by NO_3_^–^. Compared with the wild type, *MeNPF4.5* OE-22 exhibited improved growth, yield, and NUE under both low N and normal N levels, especially in the normal N treatment. However, the growth and N uptake of RNAi plants were significantly reduced, indicating poor N uptake and utilization capacity. In addition, photosynthesis and the activities of N metabolism-related enzymes (glutamine synthetase, glutamine oxoglutarate aminotransferase, and glutamate dehydrogenase) of leaves in overexpression lines were significantly higher than those in wild type. Interestingly, the RNAi line increased enzymatic activity but decreased photosynthesis. IAA content of roots in overexpressed lines were lower than that in wild type under low N level, but higher than that of wild type under normal N level. The RNAi line increased IAA content of roots under both N levels. The IAA content of leaves in the overexpression lines was significantly higher than that of the wild type, but showed negative effects on that of the RNAi lines. Thus, our results demonstrated that the MeNPF4.5 nitrate transporter is involved in regulating the uptake and utilization of N in cassava, which leads to the increase of N metabolizing enzyme activity and photosynthesis, along with the change of endogenous hormones, thereby improving the NUE and yield of cassava. These findings shed light that MeNPF4.5 is involved in N use efficiency use in cassava.

## Introduction

Cassava (*Manihot esculenta* Crantz) is one of the three major root and tuber crops in the world, and it is the sixth largest food crop globally. It is known as the “underground granary” and “king of starch” because of its importance as a source of food, feed, and industrial processing material, and it is also an important energy crop (Zhuang et al., 2011). Nitrogen (N) is the major limiting factor for cassava yield. Fertilizers are necessary for increasing storage root yields, and thus excessive amounts of N fertilizer may be applied to obtain high yields. However, the N use efficiency (NUE) has decreased from 68 to 47% over the past 50 years (Lassaletta et al., 2014), and more than half of the N applied is lost to the environment. Excessive application of N fertilizer results in leaching of N from farmlands through surface runoff and water pollution (Li et al., 2017; Kyriacou et al., 2018). Because of this, reducing the amount of N fertilizer and improving the NUE of crops have become increasingly important goals for breeders. This is also the case for cassava, where improving the efficiency of N uptake and use is of important theoretical and practical significance for improving the ecological environment, ensuring food safety, and addressing food shortages due to population growth.

Most plants use nitrate (NO_3_^–^) as the main source of N (Wang W. et al., 2018). NO_3_^–^ is a nutrient that regulates plant growth and development (Fenchel et al., 2012), and it also acts as a signaling substance regulating gene transcription, thus affecting seed germination, plant root growth, and leaf stomatal activity (Wang et al., 2012). The uptake and transport of NO_3_^–^ in the roots and its redistribution among cells are realized through NO_3_^–^ transporters (NRTs). By using isotopic tracers ^13^N and ^15^N, two NO_3_^–^ transport systems have been found in higher plants: the high-affinity transport system, which mainly functions under low external NO_3_^–^ concentrations (<0.50 mM), and the low-affinity transport system, which mainly functions under high external NO_3_^–^ concentrations (≥0.50 mM) (Miller et al., 2007; Wang Y. Y. et al., 2018; Gao et al., 2019). High- and low-affinity NRTs are encoded by the *NRT2* and *NRT1* gene families, respectively. The Nitrate Transporter 1 (NRT1) gene family got its name because it was originally found to have the function of transporting NO_3_^–^, the function of transporting dipeptides was found later and then was further classified as the PEPTIDE TRANSPORTER (PTR) family. However, several studies in the past few years confirmed that an even wider range of molecules are transported by some family members (Zhou et al., 1998; Jeong et al., 2004). Léran et al. (2014) renamed it as NPF (NRT1/PTRFAMILY) family according to its systematic evolutionary characteristics. Currently, 53 *NRT1*/*PTR* family members and seven *NRT2* family members have been found in *Arabidopsis thaliana* (Okamoto et al., 2003). AtNRT1.1 also known as CHL1 and AtNPF6.3, was the first member identified as a low-affinity transporter, but it also functions as a high-affinity transporter at low external NO_3_^–^ concentrations depending upon its phosphorylation state (Liu et al., 1999; Ho et al., 2009; Guo et al., 2014). It plays a key role in sensing and triggering many adaptive changes in response to external NO_3_^–^, such as stimulating lateral root elongation in NO_3_^–^ rich patches (Remans et al., 2006). NRT1.1 can activate these responses through many independent mechanisms, which can be uncoupled by introducing point mutations in different regions of the protein (Bouguyon et al., 2015). In addition to NO_3_^–^ uptake, NRT1.1 regulates the expression of many NO_3_^–^ responsive genes (Undurraga et al., 2017). Furthermore, NRT1.1 is a master player in the NO_3_^–^ mediated regulation of root system architecture because it stimulates the growth of lateral roots and tap roots of *Arabidopsis* (Remans et al., 2006; Fan et al., 2017; Maghiaoui et al., 2021). Some NRT1 family proteins transport NO_3_^–^ and other diverse compounds, such as nitrite (Sugiura et al., 2007), amino acids (Zhou et al., 1998), peptides (Komarova et al., 2008), and phytohormones including auxin (Krouk et al., 2010), gibberellin (Tal et al., 2016), and abscisic acid (Kanno et al., 2012), which indicates that they have versatile functions.

At present, *NRT/NPF* genes have mainly been identified in *A. thaliana* and rice, and their functions and regulatory mechanisms have been studied in detail (Liu et al., 2015; Sakuraba et al., 2021). For example, studies have shown that rice NRT1.1B is located in the cell membrane and plays an important role in the transport of NO_3_^–^ from the adventitious roots to the leaves (Hu et al., 2015; Zhao et al., 2017). Cassava is an allodiploid species (2*n* = 36) with a highly heterozygous genome, therefore, the function and expression of NRT genes in cassava deserve our attention. *MeNRT2.1* of cassava was found to be mainly expressed in the root system, and transient expression experiments in protoplasts revealed that the MeNRT2.1 protein is localized on the cell membrane (Zou et al., 2019). Ren et al. (2019) discovered that the *MeNRT2.5* gene was expressed in the roots, stems, leaves, flowers, and other organs, with relatively high expression in the roots of mature cassava plants and the leaves of tissue-cultured seedlings; and the expression of this gene was found to be inhibited by high concentrations of NO_3_^–^. Despite the progress in characterizing members of the *NRT2* gene family, there has been no report on the *NRT1* gene family in cassava. In this study, the *MeNPF4.5* (*MeNRT1.1*) gene was identified and found to be mainly expressed in cassava roots. Overexpression of this gene in cassava improved the yield of storage roots by increasing the activities of enzymes related to N metabolism and enhancing photosynthesis. In contrast, RNA interference (RNAi) significantly decreased yield, which indicated that MeNPF4.5 plays a vital role in cassava growth by regulating the metabolism and distribution of N.

## Materials and Methods

### Phylogenetic Analysis of NRT1.1

For phylogenetic analysis of NRT1.1, amino acid sequences of NRT1 from different species were obtained from GeneBank and sequence alignment was carried out using DNAMAN software (version 9.0). A phylogenetic tree based on entire amino acid sequences was constructed using the neighbor-joining method with 1000 bootstrap replicates in MEGA 7.0 (Li et al., 2018).

### Plasmid Construction and Cassava Transformation

Cassava *MeNPF4.5* (GeneBank accession No. KU361329.1) was cloned from the root cDNA of TMS60444 seedlings, which was cultured in normal condition without NO_3_^–^ induction. *MeNPF4.5* was controlled by the CaMV35S promoter. The expression cassettes was inserted into the binary vector pCAMBIA1301 containing the hygromycin phosphotransferase under the control of the CaMV 35S promoter to generate pC-35S:MeNPF4.5. And a binary expression vector p35S:MeNPF4.5 was constructed according to previous report (Vanderschuren et al., 2009). The plasmids were mobilized into *Agrobacterium tumefaciens* LBA4404, and cassava TMS60444 was used as donor plant to produce transgenic plants. Transgenic plants were produced by reported methods (Zhang et al., 2000).

### Subcellular Localization of MeNPF4.5 Protein

The open reading frame of *MeNPF4.5* without a stop codon was amplified using *MeNPF4.5* gene-specific primers (F: cagtGGTCTCacaacatgcttttcactggacttta; R: cagtG GTCTCatacaaacttgtatcaattcga cct) The PCR amplification product was cloned into the pBWA(V)HS-ccdb-GLosgfp vector to generate the MeNPF4.5-enhanced green fluorescent protein (EGFP) C-terminal fusion construct, and 35S-EGFP was used as a negative control. The recombinant plasmids were transferred into *Agrobacterium tumefaciens* strain GV3101 by electroporation and then transformed into *Nicotiana benthamiana* leaves. Two days later, EGFP fluorescence was observed at 488 nm and chloroplast fluorescence was observed at 640 nm under a confocal laser scanning microscope (C2-ER, Nikon, Japan).

### Southern Blot and qRT-PCR Analysis

Southern- blot analysis was carried out as described by Xu et al. (2012). Genomic DNA was extracted and digested with *Hind* III, separated by electrophoresis on a 0.8% (w/v) agarose gel, and then transferred to a nylon membrane with a positive charge (Roche, Shanghai, China). The *hygromycin phosphotransferase* (*HPT*) (1 kb) and *MeNPF4.5* (1.5 kb) probes were labeled with digoxigenin. Hybridization and detection were performed with the DIG-High Prime DNA Labeling and Detection Starter Kit II (Roche), according to the manufacturer’s instructions.

Total RNA was extracted from different cassava tissues (100 mg each) using RNA Plant Plus Reagent (Tiangen Biotech, Co., Beijing, China), according to the manufacturer’s instructions. cDNA was synthesized using the Prime Script™ RT Reagent Kit with gDNA Eraser (Perfect Real Time) (Takara Bio Inc., Kyoto, Japan). The specificity of the gene specific primers was verified by melting curve analysis. The cassava *Actin* gene was used as an internal control, and the 2^–ΔΔCt^ method was used to calculate relative gene expression. The primer sequences were as follows: *MeNPF4.5* (F: 5′-CCT CAA TTC CAG TGA TAC CTC TGC TTT-3′; R: 5′-GGA TTC CTG TGA TCT TCC GAA CCA AT-3′) and *MeActin* (F: 5′-CTC GTG TCA AGG TGT CGT GA-3′; R: 5′-GCC CTC TCA TTT GCT GCA AT-3′).

### Cassava Tissue Culture Experiment

The tested materials were the wild-type cassava (*Manihot esculenta* Crantz) cultivar TMS60444 (WT), overexpression lines *MeNPF4.5* OE-22 and *MeNPF4.5* OE-34, and RNAi line *MeNPF4.5* Ri-1. The stems of WT and *MeNPF4.5* transgenic cassava (with one sprout) were subcultured in 1/2 Murashige-Skoog (MS) (Murashige and Skoog, 1962) medium (Hope Bio-Technology, Qingdao, China) in a tissue culture room for 10 days. Germinated seedlings were then transferred in MS medium without N–NH_4_^+^, and the medium was supplemented with KNO_3_ as a sole N source at the concentrations as indicated in each individual experiment. Three NO_3_^–^ treatments as follows, N-free: 0 mM, low-N: 0.5 mM, and full-N: 20 mM. For N-free and low-N conditions, ion equilibrium of the medium was ensured by replacing KNO_3_ by K_2_SO_4_. The pH of the medium was adjusted to 6.0 by using NaOH. The cassava seedlings were then incubated at 26°C and 50% relative humidity with a 14 h light/10 h dark cycle. Light intensity during the day period was 250 μmol m^–2^ s^–1^. The medium contained 25 g L^–1^ sucrose and 1 g L^–1^ Gelrite. The roots were cut at the distance of 1.5 cm from root tip, stems were cut at the distance of 1.5 cm from tip, and the second expanded leaves samples were harvested 25 days after treatment with NO_3_^–^, frozen in liquid nitrogen, and stored at −80°C until further use.

Wild-type seedlings were grown in MS medium for 25 days and then transferred to hydroponic solution with 10 mM NO_3_^–^. After cultivation for 1 week, seedlings were transferred to 0.1 mM CaSO_4_ for 2 days and then to a complete nutrient solution containing 20 mM NO_3_^–^. Roots were harvested at 1, 2, 4, 6, 8, 12, and 24 h after treatment in 20 mM NO_3_^–^, frozen in liquid nitrogen, and stored at −80°C for qRT-PCR analysis.

### Root NO_3_^–^ Uptake

Wild-type and *MeNPF4.5* transgenic cassava seedlings were grown in MS medium for 25 days and then transferred to hydroponic solution with 10 mM NO_3_^–^. After cultivation for 1 week, seedlings were transferred to 0.1 mM CaSO_4_ for 2 days and then exposed to various NO_3_^–^ concentrations for 6 h. Roots were separated from shoots and measure the fresh root weight. Root NO_3_^–^ uptake were determined by disappearance of NO_3_^–^ from the nutrient solution. Their NO_3_^–^ concentration was determined by using Flow Injection Analyzer (FIASTAR 5000, Foss Analytical, Höganäs, Sweden).


$${\text{NO}}_{3}^{-}\text{uptake}=\left[\left(\mathrm{V1}\times \mathrm{C1}\right)-\left(\mathrm{V2}\times \mathrm{C2}\right)\right]/\left(\text{W}\times \text{T}\right)$$

where V1 and C1 are the volume of the system and the NO_3_^–^ concentration before experiment, and V2 and C2 are the volume of the system and the NO_3_^–^ concentration after experiment. W is fresh weight of root. T = 6 h.

### Field Experiment

#### Plant Growth Condition

Field experiment was performed in 2016 at Jiatapo, Dingxi Village, Pumiao Town, Nanning City (22°81’N, 108°63’E). The test soil was evenly flat and had uniform fertility. The chemical compositions of the cultivated soil layers before the experiment are listed in Supplementary Table 1. According to the NY/T1749-2009 Soil Fertility Diagnosis and Evaluation Method of Farmland in Southern China standards for total N (1.0 g kg^–1^) and available N (105 mg kg^–1^), the soil layers of the test plot were low in N.

A double factor split-plot design was used for field trial; the main and sub-plots were N treatment and cassava lines, respectively. There were two N treatments, low (0 kg ha^–1^, N0) and normal N (125 kg ha^–1^, N1), and the N fertilizer was urea (containing 46.7% N). A random block design was adopted with three replications. The same amounts of phosphorus (P) and potassium (K) fertilizers were used for all plots (P_2_O_5_ 48 kg ha^–1^; K_2_O 162 kg ha^–1^). Super phosphate was used as a P fertilizer, and potassium chloride was used as a K fertilizer. The P fertilizer was applied once at sowing, while the N and K fertilizers were applied thrice: 50% as a base fertilizer, 25% at 40 days after sowing, and 25% at 90 days after sowing.

For the basal fertilizer application, a 10 cm deep groove was dug at a distance of 15 cm away from the cassava stem; all the basal fertilizers were applied evenly in this groove and covered with soil. For the top dressing, the fertilizer was dissolved in water and then applied quantitatively to each plant. The size of the subplot was 10 m^2^ (1 m × 10 m), and the distance between cassava plants was 1 m × 1 m. To prevent leaching of the fertilizer from the N1 plot to the N0 plot, the two N treatment plots were separated by 1 m. The cassava plants were planted on May 10, 2016, and conventional field management methods were used during the entire growth period. Plants were harvested for agronomic trait evaluation and subsequent experiments on January 10, 2017.

#### Nitrogen Use Efficiency and Metabolism Enzyme Assays

During the harvesting of cassava, the fresh weights of leaves, stems, and storage roots of individual plants were measured. A fixed amount of each component was collected and dried at 105°C for 30 min and then further dried at 65°C to a constant weight. Tissue N concentration was determined by the micro-Kjeldahl method (Mishra et al., 2016) after digestion by concentrated H_2_SO_4_–H_2_O_2_.

The methods for calculating each indicator were as follows:

Dry matter mass of the whole plant (t ha^−1^) = dry storage root weight + dry stem weight + dry leaf weight (Kang et al., 2020).

N accumulation (g plant^−1^) = N content of the organ × dry matter mass of the organ (Kang et al., 2020).

Transport index (%) = Shoot N content/(Root N content + Shoot N content) × 100 (Léran et al., 2013).

Nutilization efficiency (kg kg^−1^) = Plant dry weight/N accumulation (Kang et al., 2020).

N recovery efficiency (kg kg^−1^) = (N uptake with fertilizer – N uptake with nofertilizer)/N application rate (Peng et al., 2006).

Partial factor productivity of N fertilizer (kg kg^−1^) = Storage root yield/N application rate (Peng et al., 2006).

The fifth expanded leaves from plants grown for 3 months were collected with three replications. The midrib was removed, and the sample was cut and mixed evenly to determine the activity of key enzymes involved in nitrogen metabolism. Nitrate reductase (NR) activity was measured using a kit from the Nanjing Jian Cheng Bioengineering Institute (Nanjing, China), according to the manufacturer’s instructions. The activities of glutamine synthetase (GS), glutamine oxoglutarate aminotransferase (GOGAT), and glutamate dehydrogenase (GDH) were determined using the method described by Huang et al. (2015).

#### Leaf Photosynthesis and Chlorophyll Fluorescence Parameters Analysis

The photosynthetic activity of the fifth expanded leaves from plants grown for 3 months was measured with a 6400XT (LI-COR, Lincoln, Nebraska, United States) photosynthesis system on a sunny day from 9:00 to 11:00 in the morning. The net photosynthetic rate (Pn), intercellular CO_2_ concentration (Ci), stomatal conductance (Gs), and transpiration rate (Tr) were determined. The photosynthetic photon flux density was 1,200 μmol m^–2^s^–1^.

The following chlorophyll fluorescence parameters were recorded from leaves at the same position as those used for photosynthetic parameter measurements using an imaging chlorophyll fluorimeter (Walz Imaging PAM, Walz GmbH, Effeltrich, Germany): Fv/Fm (PS II maximum quantum yield of photosynthesis), Y(II) (PS II actual quantum yield of photosynthesis), qP (photochemical quenching coefficient), and qN (non-photochemical quenching coefficient). The measurements were conducted at room temperature (25°C) using the standard saturated light mode. The actinic light intensity was 10 μmol m^–2^ s^–1^. Prior to detection, leaves were adapted to darkness for 30 min.

### Endogenous Hormone Analysis

Cassava seedlings were cultivated in tissue culture under different N conditions (0.5 and 20 mM NO_3_^–^) for 25 days, and then the endogenous hormone content in the roots (excised from 1.5 cm of primary root tips) and leaves (the second expanded leaves) of WT and transgenic plants were analyzed. The extraction, purification and determination of endogenous hormones levels were assayed by an indirect enzyme-linked immunosorbent assay (ELISA) technique, which performed with a kit from the Beijing Benongda Tianyi Biotechnology Co. (Beijing, China) according to the manufacturer’s instructions.

### Statistical Analysis

Data from at least three biological replicates are presented as the mean ± SE. Analysis of variance (ANOVA) followed by independent sample Student’s *t*-test was performed using SPSS software version 22.0 (IBM Corp., Armonk, NY, United States). *P* < 0.05 was considered statistically significant.

## Results

### Phylogenetic Analysis of NRT1.1 and MeNPF4.5 Is a Plasma Membrane Localized Protein

**FIGURE 1:**
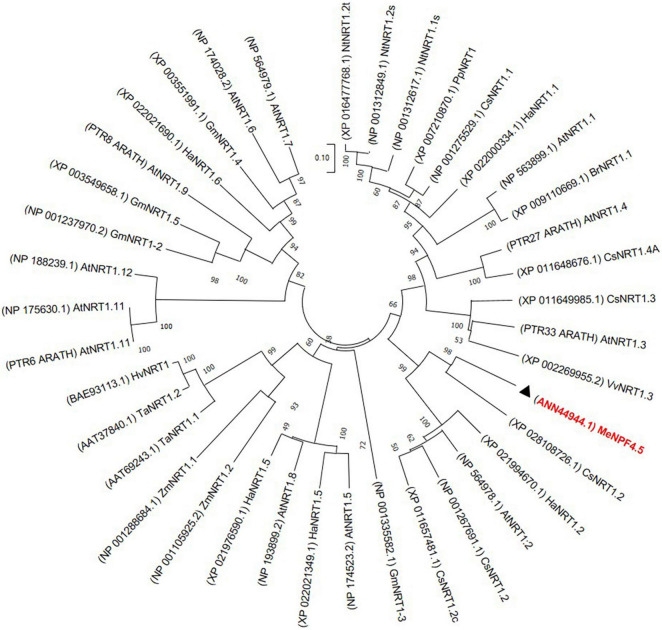
Phylogenetic analysis of the MeNPF4.5 protein and other NO_3_^–^ transporters (NRTs). The amino acid sequences were aligned using ClustalW software and the phylogeny was constructed using the neighbor-joining method with 1000 bootstrap replicates in MEGA7.

Phylogenetic analysis showed that MeNPF4.5 is closely related to CsNRT1.2 of *Camellia sinensis* and HaNRT1.2 of *Helianthus annuus.* These results showed that MeNPF4.5 is a member of the NRT1/NPF subfamily (Figure 1).

**FIGURE 2:**
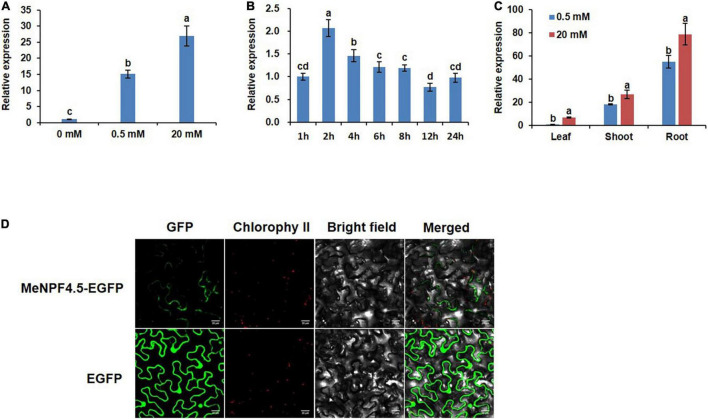
Tissue-specific expression pattern and subcellular localization of the MeNPF4.5. **(A)** Relative expression of *MeNPF4.5* in the roots of plants grown under different N conditions for 25 days. **(B)** Expression levels of *MeNPF4.5* in N-starved roots at different times after N induction. Wild-type (WT) seedlings were grown in 10 mM NO_3_^–^ for 20 days and under N starvation for 2 days, then were transferred to 20 mM NO_3_^–^. **(C)** Expression of *MeNPF4.5* in various tissues of cassava. Cassava seedlings were cultivated under different N conditions (0.5 and 20 mM NO_3_^–^) in tissue culture for 25 days. Values are the means of three biological replicates ± SE. Lowercase letters above the bars (a–d) represent significant differences between treatments as determined by Student’s *t*-test (*P* < 0.05). **(D)** Subcellular localization of the MeNPF4.5-EGFP fusion protein in tobacco leaf epidermal cells. From left to right, GFP fluorescence, chlorophyll fluorescence, bright field, and merged images are shown. Bars = 20 μm.

A 35S-MeNPF4.5-EGFP fusion protein construct was used to determine the subcellular localization of MeNPF4.5, and 35S-EGFP served as control. The constructs were transiently transformed into leaf cells of *Nicotiana* using agroinfiltration. MeNPF4.5-EGFP was expressed in the plasma membrane, whereas EGFP was detected not only in the plasma membrane, but also in the cytoplasm and nucleus (Figure 2D). These results indicated that MeNPF4.5 is a transmembrane transport protein.

### Expression of MeNPF4.5 Is Tissue Specific

To analyze the expression of *MeNPF4.5* in response to different N levels, young cassava seedlings were grown in N-free (0 mM NO_3_^–^), low-N (0.5 mM NO_3_^–^), and full-N (20 mM NO_3_^–^) media. We found that the expression level of *MeNPF4.5* in roots significantly decreased under N-free conditions. Compared with the N-free control where *MeNPF4.5* was expressed at the lowest levels, *MeNPF4.5* expression in roots was increased by 15.1-fold under low-N and 27.0-fold under full-N, which indicated that *MeNPF4.5* expression was induced by NO_3_^–^ (Figure 2A).

*MeNPF4.5* expression in roots was also analyzed with a time-course experiment. After NO_3_^–^ induction, the expression of *MeNPF4.5* increased rapidly and reached a peak at 2 h. Expression then decreased gradually and stabilized at 6 h, but then decreased again, reaching the lowest level at 12 h before increasing again at 24 h (Figure 2B). Analysis of expression in different tissues showed that *MeNPF4.5* was expressed in stems and leaves in addition to roots; and the expression level was highest in roots under both 0.5 and 20 mM NO_3_^–^, with expression levels 54.9- and 11.4-times higher than those in leaves, respectively (Figure 2C).

### Identification of MeNPF4.5 Transgenic Cassava Plants

**FIGURE 3:**
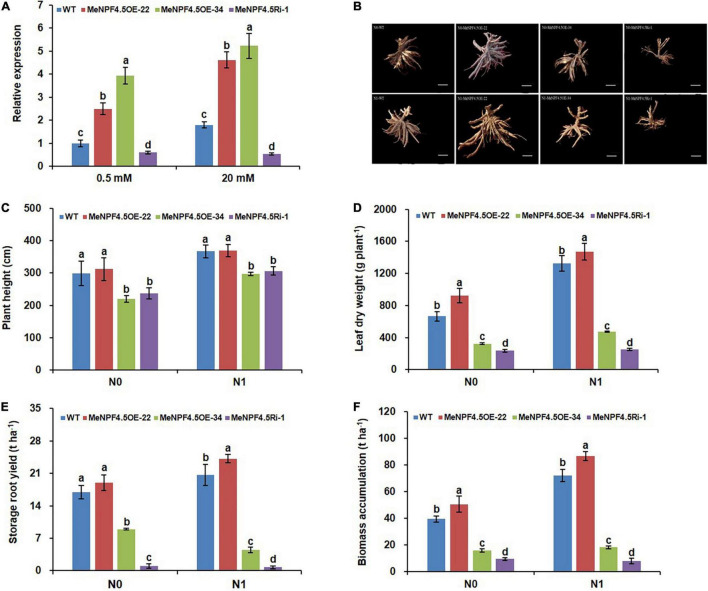
Molecular identification and analysis of agronomic traits of WT and transgenic plants. **(A)** Real-time RT-PCR analysis of *MeNPF4.5* gene expression in the roots of WT and transgenic plants under 0.5 and 20 mM NO_3_^–^. *MeNPF4.5* OE, *MeNPF4.5* overexpression; *MeNPF4.5* Ri, *MeNPF4.5* RNA interference. The *Actin* gene was used as an internal control. **(B–F)** Agronomic traits of WT and transgenic plants grown in the field for 8 months under different N0 (0 kg ha^–1^) and N1 (125 kg ha^–1^) conditions. **(B)** Storage roots of cassava plants grown in the field. N0 treatment (upper panel), N1 treatment (lower panel); from left to right, WT, *MeNPF4.5* OE-22, *MeNPF4.5* OE-34, and *MeNPF4.5* Ri-1 images are shown. Bars = 10 cm. **(C)** Plant height (*n* = 10). **(D)** Leaf dry weight (*n* = 5). **(E)** Storage root yield (*n* = 5). **(F)** Biomass accumulation (*n* = 5). Values are means ± SE. Lowercase letters above the bars (a–d) represent significant differences between treatments as determined by Student’s *t*-test (*P* < 0.05). N0, low-N treatment, 0 kg ha^–1^; N1, normal N treatment, 125 kg ha^–1^.

To investigate the function of *MeNPF4.5*, the 3 transgenic lines (overexpression lines *MeNPF4.5* OE-22 and *MeNPF4.5* OE-34, and RNAi line *MeNPF4.5* Ri-1) were constructed. Southern blot analysis verified that the exogenous *MeNPF4.5* gene had been integrated into the genomes of *MeNPF4.5* OE-22 and *MeNPF4.5* OE-34 (Supplementary Figure 1), which confirmed that the overexpression lines *MeNPF4.5* OE-22 with a single copy of the transgenic construct and *MeNPF4.5* OE-34 with two copies. Quantitative RT-PCR was used to assess the levels of *MeNPF4.5* RNA in these lines. As shown in Figure 3A, the expression levels of *MeNPF4.5* in *MeNPF4.5* OE-22 and *MeNPF4.5* OE-34 were significantly higher than those in the WT under both N conditions, and the expression level in the Ri-1 line was decreased by 39.7 and 70.4% under low and normal N conditions, respectively. Therefore, these three lines were used for further analysis.

### MeNPF4.5 Overexpression Enhances the Yield of Storage Roots

Lush foliage is the basis for healthy and high-yielding crops. Field evaluation of *MeNPF4.5* transgenic cassava showed that the two transgenic overexpression lines *MeNPF4.5* OE-22 and *MeNPF4.5* OE-34 and the RNAi line *MeNPF4.5* Ri-1 exhibited significant differences from WT in both the aerial and underground parts (Figure 3). Three of the four agronomic traits examined, namely leaf dry weight, storage root yield, and biomass accumulation, were significantly higher in *MeNPF4.5* OE-22 than in WT under both low and normal N conditions, whereas these trait values were lower in *MeNPF4.5* Ri-1. There was no significant difference in plant height between *MeNPF4.5* OE-22 and WT, but both lines were significantly taller than *MeNPF4.5* OE-34 and *MeNPF4.5* Ri-1 under both low and normal N conditions. These results demonstrated that *NRT1.1* overexpression can improve plant growth under low or normal N conditions. However, no improvement in agronomic traits was observed for the *MeNPF4.5* OE-34 line; all four traits examined were lower than those in WT and *MeNPF4.5* OE-22, but higher than those in *MeNPF4.5* Ri-1.

### MeNPF4.5 Improves N Uptake, N Translocation, N Accumulation, and Nitrogen Use Efficiency

**FIGURE 4:**
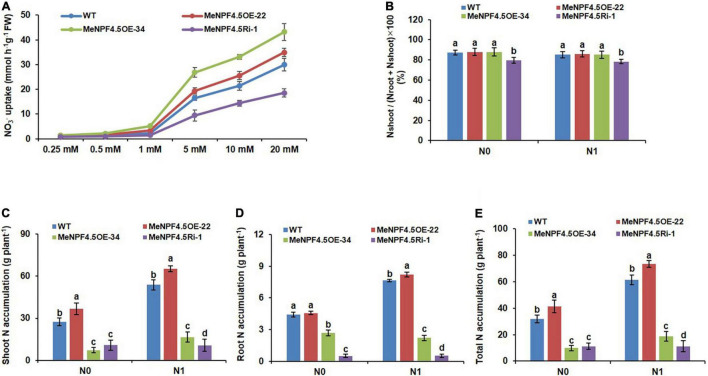
Nitrogen uptake, tanslocation and accumulation in WT and *MeNPF4.5* transgenic cassava plants. **(A)** Root NO_3_^–^ uptake in WT and *MeNPF4.5* transgenic cassava plants. WT and *MeNPF4.5* transgenic cassava plants grown in 20 mM KNO_3_ for 25 days and then deprived of N for 3 days. N-starved plant roots were then exposed to various NO_3_^–^ concentrations for 6 h, uptake of NO_3_^–^ were measured by disappearance of NO_3_^–^ from the nutrient solution. **(B)** Nitrate translocation in WT and *MeNPF4.5* transgenic cassava plants. **(C–E)** N accumulation in wild-type (WT) and *MeNPF4.5* transgenic cassava plants harvested from the field. **(C)** Shoot N accumulation. **(D)** Root N accumulation. **(E)** Total N accumulation. Values are the means of three biological replicates ± SE. Lowercase letters above the bars (a–d) represent significant differences between treatments as determined by Student’s *t*-test (*P* < 0.05). N0, low-N treatment, 0 kg ha^–1^; N1, normal N treatment, 125 kg ha^–1^.

To determine the MeNPF4.5 function in NO_3_^–^ uptake by roots, we measured the NO_3_^–^ uptake of cassava roots. The results showed that the NO_3_^–^ uptake of WT and transgenic cassava roots increased gradually with increasing of NO_3_^–^ concentration. The NO_3_^–^ uptake was significantly higher in overexpression lines (*MeNPF4.5* OE-22 and *MeNPF4.5* OE-34) than those in the WT at relatively higher (1–20 mM) NO_3_^–^ concentration. However, no significant difference was found at relatively lower (0.25 mM) NO_3_^–^ concentrations. By contrast, the NO_3_^–^ uptake was significantly lower in *MeNPF4.5* Ri-1 line than that in the WT at all tested NO_3_^–^ concentrations (Figure 4A). Therefore, it seems that MeNPF4.5 is involved in the function of NO_3_^–^ uptake.

Transport index was used as an indicator of NO_3_^–^ translocation from roots to shoots (Figure 4B). Translocation of NO_3_^–^ from roots to shoots between WT and transgenic lines, showing that the *MeNPF4.5* Ri-1 plants have lower N enrichment in the shoots compared to those of WT and OE transgenic plants under both N conditions. However, N enrichment had no remarkable between OE transgenic plants (*MeNPF4.5* OE-22 and *MeNPF4.5* OE-34) and WT under the N0 and N1 conditions, indicating that the translocation of NO_3_^–^ to shoots is slower when *MeNPF4.5* gene expression is suppressed, thus it can be seen that the MeNPF4.5 activity affects the NO_3_^–^ translocation from roots to shoots.

As *NRT1.1* is one of the NRT genes involved in NO_3_^–^ uptake in roots, we analyzed the N accumulation of transgenic lines and WT plants. N accumulation is equal to the N content in each part of the plant multiplied by the biomass. As shown in Figures 4C–E, the amounts of shoot and whole-plant N accumulation in *MeNPF4.5* OE-22 under low-N and normal N treatments were significantly higher than those in the WT, by 33.7% (N0) and 36.5% (N1) for shoots and by 29.5% (N0), 19.5% (N1) for the whole plant, but N accumulation was significantly lower in the *MeNPF4.5* Ri-1 line than in the WT. The amounts of N accumulation in storage roots of *MeNPF4.5*OE-22 were similar to those in WT under low-N conditions, but higher than those in WT under normal N conditions, which verified that *NRT1.1* was induced by high NO_3_^–^ (≥0.50 mM). All these results show that *NRT1.1* enhances NO_3_^–^ uptake and accumulation in cassava.

The NUE of crops is related to the N uptake efficiency and N utilization efficiency, but their contributions to N efficiency remain controversial (Bogard et al., 2013). Our results indicated that there were significant differences in N utilization efficiency (NUtE), N recovery efficiency (NRE), and the partial factor productivity of N (PFPN) of the different transgenic cassava lines. As shown in Table 1, there were no significant differences in NUtE (under N0) and NRE between *MeNPF4.5* OE-22 and WT, but NUtE (under N1) and PFPN of *MeNPF4.5* OE-22 were significantly higher than those of the WT (by 7.6 and 16.8%, respectively). The NUtE under both N levels, NRE, and PFPN of *MeNPF4.5* Ri-1 were significantly lower than those of the WT (reduced by 36.4, 130.1, and 94.7%, respectively). *MeNPF4.5* OE-34 had a lower NUtE (under N1), NRE, and PFPN than WT, but NUtE under N0 conditions was significantly higher than that in the WT. These results indicated that the *MeNPF4.5* gene expression level could severely affect the activity of NPF4.5 transporter in uptake and utilization of N under different N levels.

**TABLE 1:** Differences in nitrogen uptake and utilization of wild-type and *MeNPF4.5* transgenic cassava.

Lines	N utilization efficiency		N recovery efficiency (kg kg^–1^)	Partial factor productivity of applied N (kg kg^–1^)
	(kg kg^–1^)			
	N0	N1		
WT	124.73 ± 5.93b	109.72 ± 4.71b	2.78 ± 0.15a	166.34 ± 3.06b
*MeNPF4.5* OE-22	127.15 ± 1.77b	118.08 ± 1.58a	2.68 ± 0.13a	194.28 ± 1.24a
*MeNPF4.5* OE-34	179.57 ± 9.42a	99.29 ± 1.09c	0.79 ± 0.04b	36.31 ± 2.62c
*MeNPF4.5* Ri-1	91.64 ± 3.27c	75.30 ± 1.8d	0.02 ± 0.00c	6.12 ± 0.83d

*N0, low-N treatment, 0 kg ha^–1^; N1, normal N treatment, 125 kg ha^–1^. Values are the means of three biological replicates ± SE. Values labeled with different letters represent significant differences between respective treatments by Student’s t-test (P < 0.05).*

### MeNPF4.5 Increases the Activities of Enzymes Related to N Metabolism in Cassava Leaves

**FIGURE 5:**
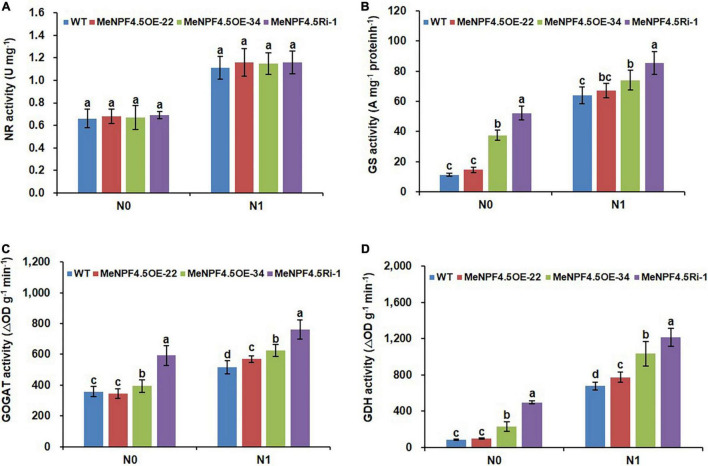
Activity of N metabolism enzymes. Activities of NR **(A)**, GS **(B)**, GOGAT **(C)**, and GDH **(D)** in the leaves of WT and *MeNPF4.5* transgenic cassava harvested from the field. Values are the means of three biological replicates ± SE. Lowercase letters above the bars (a–d) represent significant differences between treatments as determined by Student’s *t*-test (*P* < 0.05). N0, low-N treatment, 0 kg ha^–1^; N1, normal N treatment, 125 kg ha^–1^.

The N absorbed by crop roots must be assimilated into organic matter through N metabolism enzymes before it can be used by the plant. The activity of key N metabolism enzymes can directly reflect the strength of N metabolism in crops (Andrews et al., 2004). The activities of enzymes involved in N metabolism, namely NR, GS, GOGAT, and GDH, were higher in WT and all the transgenic plants under the N1 treatment than under the N0 treatment, which showed that high N promoted the activities of these enzymes (Figure 5). The activities of all enzymes except for NR were higher in *MeNPF4.5* OE-34 and *MeNPF4.5* Ri-1 than in *MeNPF4.5* OE-22 and the WT under both the N0 and N1 conditions; at the same time, the activities of these enzyme were significantly higher in *MeNPF4.5* OE-22 than in WT under the N1 conditions.

### MeNPF4.5 Increases Photosynthesis

**FIGURE 6:**
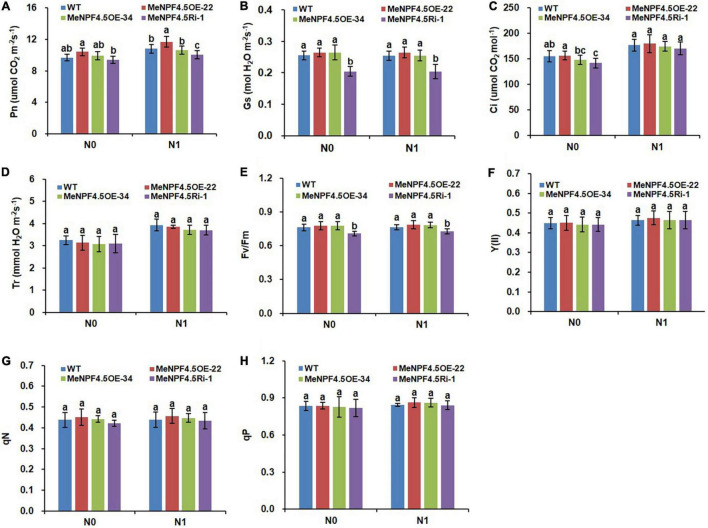
Photosynthetic and chlorophyll fluorescence parameters of WT and *MeNPF4.5* transgenic cassava plants. **(A)** Photosynthetic rate (Pn). **(B)** Stomatal conductance (Gs). **(C)** Intercellular CO_2_ concentration (Ci). **(D)** Transpiration rate (Tr). **(E)** Fv/Fm. **(F)** Y(II). **(G)** qN. **(H)** qP. Values are the means of five biological replicates ± SE. Lowercase letters above the bars (a-d) represent significant differences between treatments as determined by Student’s *t*-test (*P* < 0.05). N0, low-N treatment, 0 kg ha^–1^; N1, normal N treatment, 125 kg ha^–1^.

Photosynthesis is the physiological basis of crop growth and yield formation, and 90–95% of crop dry matter accumulation originates from photosynthetic products. As shown in Figures 6A–D, there was no significant difference in photosynthetic parameters, namely Gs, Ci, and Tr, between *MeNPF4.5* OE-22, *MeNPF4.5* OE-34, and WT under both N levels. The Pn of *MeNPF4.5* OE-22 was 8.4% higher compared with that of WT under the N1 conditions. The Pn and Gs of *MeNPF4.5* Ri-1 were significantly lower than those of WT under both N levels.

Chlorophyll fluorescence is a direct indicator of plant physiology and reflects the photochemical process and its efficiency. As photosynthesis was altered by the *MeNPF4.5* gene (Figures 6A–D), we wanted to evaluate the differences in chlorophyll fluorescence parameters between the transgenic lines and WT. The Fv/Fm values of the leaves of *MeNPF4.5* Ri-1 plants were significantly lower compared with those of WT and OE transgenic plants under the N0 and N1 conditions (Figures 6E–H), but other chlorophyll fluorescence parameters did not significantly differ between the transgenic plants and WT.

### MeNPF4.5 Regulates Endogenous Hormone Levels in Cassava

**FIGURE 7:**
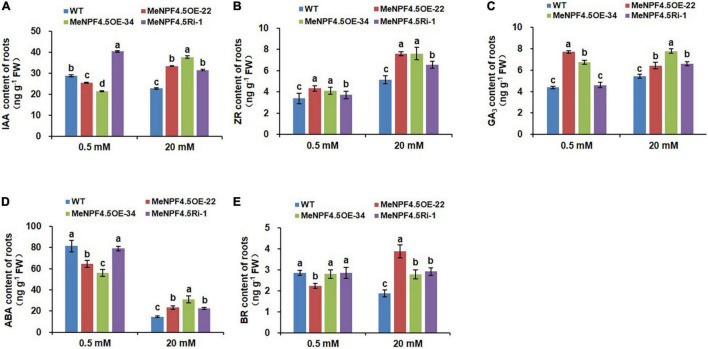
Endogenous hormone levels in roots of WT and *MeNPF4.5* transgenic cassava plants cultivated in tissue culture for 25 days. **(A–E)** contents of indoleacetic acid (IAA) **(A)**, zeatin-riboside (ZR) **(B)**, gibberellic acid (GA_3_) **(C)**, abscisic acid (ABA) **(D)**, and brassinolide (BR) **(E)**. Values are the means of three biological replicates ± SE. Lowercase letters above the bars (a–d) represent significant differences between treatments as determined by Student’s *t*-test (*P* < 0.05).

**FIGURE 8:**
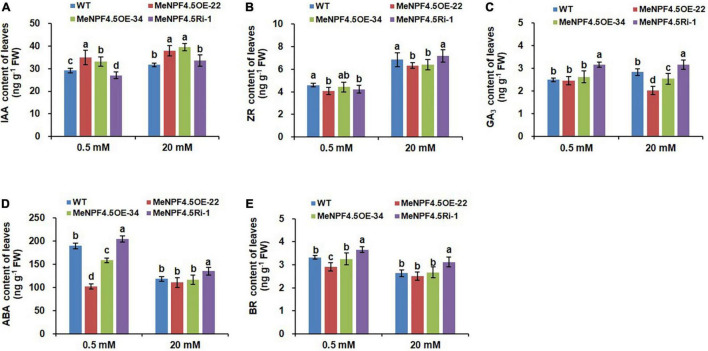
Endogenous hormone levels in leaves of WT and *MeNPF4.5* transgenic cassava plants cultivated in tissue culture for 25 days. **(A–E)** Contents of indoleacetic acid (IAA) **(A)**, zeatin-riboside (ZR) **(B)**, gibberellic acid (GA_3_) **(C)**, abscisic acid (ABA) **(D)**, and brassinolide (BR) **(E)**. Values are the means of three biological replicates ± SE. Lowercase letters above the bars (a–d) represent significant differences between treatments as determined by Student’s *t*-test (*P* < 0.05).

Significant changes were also detected in endogenous hormones, namely indoleacetic acid (IAA), zeatin-riboside (ZR), gibberellic acid (GA_3_), abscisic acid (ABA), and brassinosteroid (BR), in the roots and leaves of transgenic cassava (Figures 7, 8). In roots, compared with WT, the levels of IAA were 11.8 and 25.8% lower in *MeNPF4.5* OE-22 and OE-34, respectively, and 39.9% higher in *MeNPF4.5* Ri-1 under low-N conditions (Figure 7). In contrast, under the full-N conditions the levels of IAA in transgenic plants (*MeNPF4.5* OE-22, *MeNPF4.5* OE-34, and *MeNPF4.5* Ri-1) were 46.7, 65.2, and 38.2% higher than those in WT, respectively. For ZR and GA_3_, very similar patterns were observed in transgenic plants under both N levels. All the transgenic lines had a significantly higher ZR and GA_3_ contents than WT under both N conditions. Analysis of ABA revealed decreases of 20.5% in *MeNPF4.5* OE-22 and 31.0% in *MeNPF4.5* OE-34 compared with WT. ABA levels in *MeNPF4.5* Ri-1 plants did not significantly differ from those in WT under low-N conditions. Under full-N conditions, the ABA content was higher compared with that in WT in all transgenic plants. Compared with WT plants, there was a 21.5% decrease of BR in *MeNPF4.5* OE-22 and no significant difference between WT and the *MeNPF4.5* OE-34 and *MeNPF4.5* Ri-1 lines under low-N conditions. However, BR levels were higher in the three transgenic lines under full-N conditions. These results suggest that the *MeNPF4.5* gene alters plant endogenous hormone levels in transgenic plants.

Analysis results on content of endogenous hormone in leaves showed that IAA in *MeNPF4.5* OE-22 and MeNPF4.5 OE-34 were higher than those in WT under both N conditions, and IAA levels in *MeNPF*4.5 Ri-1 plants did not significantly differ from those in WT under full-N conditions but significantly lower than those in WT under low-N conditions (Figure 8). Compared with WT plants, there were significantly lower ZR contents in *MeNPF4.5* OE-22 and *MeNPF4.5* Ri-1 lines under low-N conditions. ZR levels in *MeNPF4.5* OE-34 plants did not significantly differ from that in WT under low-N conditions. Under full-N conditions, the ZR content was lower compared with that in WT in the overexpression lines. Meanwhile, the overexpression lines *MeNPF4.5* OE-22 and *MeNPF4.5* OE-34 had a significantly lower GA_3_ contents than WT under full- N conditions. And the *MeNPF4.5* Ri-1 lines had a significantly higher GA_3_ contents than those in WT under both N conditions. For ABA and BR, similar patterns were observed in transgenic plants under both N levels. ABA and BR levels in *MeNPF4.5* Ri-1 plants was higher compared with those in WT and in the overexpression lines under both conditions. And the overexpression lines *MeNPF4.5* OE-22 and *MeNPF4.5* OE-34 had a significantly lower ABA and BR contents than those in WT under both N conditions. These results also suggest that the modulation of *MeNPF4.5* gene expression alters plant endogenous hormone levels in transgenic plants.

## Discussion

*NRT1.1*/*NPF6.3* (*CHL1*) was the first NRT gene discovered in plants, and it also was found to function not only in NO_3_^–^ uptake (Tsay, 1993) but also in NO_3_^–^ translocation from roots to shoots (Léran et al., 2014). At present, research on *NRT1.1* has mainly focused on the model plant *Arabidopsis* and the food crops rice (Hu et al., 2015; Fan et al., 2017; Wang Y. Y. et al., 2018) and wheat (Guo et al., 2014). Little information regarding this family is available in cassava, an important crop in much of the world. In this study, we investigated the spatiotemporal expression pattern and the function of the *MeNPF4.5* gene in cassava. Our data clearly showed that *MeNPF4.5* was barely detectable under N deficiency conditions, but its expression was maintained a high level under N sufficient conditions. This suggests that *MeNPF4.5* expression could be induced by NO_3_^–^ just as *AtNRT1.1* is in *Arabidopsis* (Lejay et al., 1999; Liu et al., 1999). Once N-starved cassava plants were exposed to NO_3_^–^, *MeNPF4.5* expression first increased and then decreased (Figure 2A). It can be seen that *MeNPF4.5* responds to NO_3_^–^ in the short term like many other plants *NRT1.1* (Cárdenas Navarro et al., 1998; Okamoto et al., 2003; Wang et al., 2007). In addition to being expressed in roots, *AtNRT1.1* is also expressed in shoots, young leaves, and developing flower buds of *Arabidopsis*, but it is more strongly expressed in roots (Miller et al., 2007; Sakuraba et al., 2021). Consistent with the expression pattern of *NRT1.1* in *Arabidopsis*, *MeNPF4.5* is expressed in roots, stems, and leaves of cassava plants, and it is predominantly expressed in roots. We also conducted subcellular localization studies on the MeNPF4.5 protein and found that it was located on the plasma membrane, which was consistent with the findings of previous studies on NRT1.1 (Hu et al., 2015; Zhao et al., 2017).

Nitrate affects all aspects of plant physiology including metabolism, resource allocation, growth, and development, and its accumulation and translocation are critical to dry matter production. NRTs are not only involved in the uptake and transport of NO_3_^–^ in roots, but also in the redistribution of NO_3_^–^ among cells. The influence of the expression of NRTs on the N uptake and NUE of crops are important topics in breeding research. For example, Hu et al. (2015) demonstrated that overexpression of indica-type *OsNRT1.1B* can potentially improve the NUE of the japonica variety Zhonghua. Furthermore, a recent study showed that 35S promoter- driven expression of *OsNRT1.1A* improves the grain yield of transgenic rice plants (Wang W. et al., 2018). Overexpression of the OsNRT1.2 in the rice cultivar ‘Wuyunjing 7’ resulted in a biomass increase under a high concentration of NO_3_^–^ (Ma et al., 2011). These results suggest an intimate relationship between the function of *NRT1* and plant growth. To evaluate the effect of the *MeNPF4.5* gene on cassava growth, we analyzed the yield and biomass of transgenic lines under two different N levels in a field test. Our results revealed differences between the overexpression lines and RNAi line; the yield and biomass of the overexpression lines were higher than those of the RNAi line. Compared with the WT, the growth of cassava was promoted in the overexpression line *MeNPF4.5* OE-22 under both low and normal N conditions, while growth of the RNAi line *MeNPF4.5*Ri-1 was inhibited (Figure 3). This finding is consistent with the results of previous studies and shows that enhanced *MeNPF4.5* expression could improve growth in cassava under both low and normal N conditions. However, another *MeNPF4.5* overexpression line, *MeNPF4.5* OE-34, exhibited inhibited growth compared with WT and *MeNPF4.5* OE-22, although its growth was superior to that of *MeNPF4.5*Ri-1. This might be related to the high expression of *MeNPF4.5* in this line, which may affect the balance between N uptake and utilization; or maybe *MeNPF4.5* OE-34 has two copy of the transgenic construct, which might lead to the silence of target gene and thus affect plant growth (Vaucheret et al., 1998; Tenea et al., 2008; Ahlawat et al., 2014).

Nitrogen use efficiency can be simply defined as the yield of grain per unit of N available in the soil. NUE depends on N uptake efficiency and internal utilization efficiency, and N uptake-and-utilization efficiency reflects the capacity of crops to absorb N and transport it to the organs (Moll et al., 1982; Chen and Ma, 2015). Therefore, here we examined the association between N uptake, translocation, NUtE, and growth. *OsNRT1.1B* was reported to increase the NUtE of rice by 30% (Duan and Zhang, 2015; Hu et al., 2015), and Chen and Ma (2015) reported that *OsNRT1.1B* improves NUE in rice by enhancing root NO_3_^–^ uptake. Here we found that *MeNPF4.5* enhanced N uptake, translocation, and accumulation in cassava; NUtE, NRE, and PFPN were improved in *MeNPF4.5* overexpressing plants but were significantly lower in the *MeNPF4.5* Ri-1 line compared with WT (Table 1). It is inferred that the higher yields of the overexpression lines *MeNPF4.5* OE-22 and *MeNPF4.5* OE-34 may be due to their high N uptake and transport capabilities, while the yield of *MeNPF4.5* Ri-1 decreased because of its reduced N uptake and allocation.

N is absorbed by cassava in the form of NO_3_^–^, and then it needs to be assimilated mainly in shoot part into organic N through the catalytic action of several enzymes. Studies have found that when NO_3_^–^ addition to N-starved seedlings, all genes known to be directly required for NO_3_^–^ assimilation were strongly induced, including N metabolism enzyme gene (Gowri et al., 1992; Wang et al., 2003; Scheible et al., 2004). Hu et al. (2009) also confirmed that NO_3_^–^ assimilation genes are regulated by NO_3_^–^, and these genes are expressed at low levels in the absence of NO_3_^–^ and are rapidly induced in its presence (priming effect). Much evidence suggests that NR and GS are the key enzymes for N assimilation (Thomsen et al., 2014). For most plants, there is a positive correlation between the amount of NO_3_^–^ absorbed by the roots from the soil and NR activity (Andrews et al., 2004). Meanwhile, GS enzyme activity and stability are positively correlated with substrate abundance (Andrews et al., 2004). Other enzymes responsible for N assimilation in addition to NR and GS, such as GOGAT, GDH, and asparagine synthetase, have also been reported to play important roles in N metabolism and improvement of NUE in plants (Ameziane et al., 2000; Wong et al., 2004; Yamaya and Kusano, 2014). In this study, we found that the activity of nitrogen metabolism enzymes in the same line under N1 level was higher than that under N0 level, indicating that N metabolism enzyme were induced by NO_3_^–^. The GS, GOGAT, and GDH activities in *MeNPF4.5* OE-34 and *MeNPF4.5* Ri-1 were higher than those in *MeNPF4.5* OE-22 and the WT under both the N0 and N1 conditions, but there was no significant difference in NR activity. Although the activities of these enzymes were higher in *MeNPF4.5* OE-34 and *MeNPF4.5* Ri-1 than in the WT, the growth of these plants was inhibited and N accumulation was lower. Studies have shown that compared with the WT, transgenic rice that overexpress GS1 (Tabuchi et al., 2007) show no difference in growth phenotype and have lower yields. Transgenic tobacco plants that overexpress GS2 show limited growth and leaf yellowing (Thomsen et al., 2014). Some studies suggest that these phenomena may be the result of feedback inhibition of nitrogen metabolites (Vincentz and Caboche, 1991). The results of this study may also related to this phenomenon.

Previous studies found that there is a significant positive correlation between photosynthesis and crop yield and NUE (Zhang et al., 2017). However, some studies have pointed out that the substantial increase in crop yield achieved in the past few decades has not been accompanied by a significant increase in leaf Pn. The yield gain is mainly attributed to an increase in leaf area index and biological yield (Sharma-Natu and Ghildiyal, 2005). In this study, the leaf dry weight and Pn in *MeNPF4.5* OE-22 were significantly higher compared with those in WT, while these two traits were significantly lower in the RNAi line. The Fv/Fm values in the RNAi line were also lower. Therefore, it is speculated that the difference in leaf area index and photosynthetic parameters may underlie the difference in NUE and yield between *MeNPF4.5* OE-22 plants and *MeNPF4.5* Ri-1 and WT plants.

Hormones play an important role in regulating various metabolic, growth, and development processes, and maintaining a dynamic balance under normal physiological conditions. Lots of plant hormones are transported by some NRT proteins and conversely regulate NRT gene expression (Zhao et al., 2021). NRT1.1 in roots can transport NO_3_^–^ and auxin, but preferentially transports NO_3_^–^ (Krouk et al., 2010; Bouguyon et al., 2015). Hormone metabolism appears transcriptional reprogramming and sensing as one of the early response to NO_3_^–^ addition (Scheible et al., 2004), while higher supply of NO_3_^–^ decreased IAA concentrations in phloem exudates, which affected root growth (Bhalerao et al., 2002; Tian et al., 2008; Liu et al., 2010). Krouk et al. (2010) propose that NRT1.1 represses lateral root growth at low NO_3_^–^ availability by promoting basipetal auxin transport out of these roots. In this study, we had the similar results, the IAA content in roots of the two overexpression lines was significantly lower compared with that of WT under low-N conditions, was higher in roots of RNAi line, which might be resulted from MeNPF4.5 promoting basipetal auxin transport and lowering auxin accumulation in the roots in OE plants, while the transport ability was inhibited in RNAi lines. Under full-N conditions, it was speculated that MeNPF4.5 performed the NO_3_^–^ transport function, which could lead to higher IAA content in roots of the transgenic lines than in that of WT. In addition, our results showed that the content of IAA in leaves of overexpressed lines remained at a high level under both N levels, which may be related with a higher translocation of N toward the aerial parts associated with more production of IAA. Previous studies found that the change of IAA levels in the root was closely related to NO_3_^–^ mediated root growth, however, the latter process could not be explained exclusively by the former, due to NO_3_^–^ has a comprehensive effect on other phytohormones, such as cytokinin (Beck, 1996; Takei et al., 2001) and ethylene (Singh et al., 2000), which also exert significant effects on root development. The changes of other hormones contents (ZR, GA_3_, ABA, and BR) were also detected in roots and leaves of transgenic plants and WT under two N levels, in previous report, Tian et al. (2005) also discovered that cytokinin concentrations in roots enhanced with increasing NO_3_^–^ supply. Therefore, it is supposed that IAA might synergistically interact with ZR, GA_3_, ABA, and BR to affect the growth of cassava plants. And further study is required to elucidate the interaction among these hormones on NO_3_^–^ mediated cassava growth.

In summary, *MeNPF4.5* is mainly expressed in cassava roots, and its expression is induced by NO_3_^–^. Overexpressing *MeNPF4.5* in cassava promoted growth and improved the NUE and yield. The main reasons for these effects may be increased photosynthesis and N metabolism enzyme activity. Down-regulation of *MeNPF4.5* expression resulted in inhibition of cassava growth and reduction of NUE, causing a decrease in biomass and yield. Overall, our study reveals the role of MeNPF4.5 in N uptake and utilization, which has not been previously reported, and shows that this NRT may have potential breeding value.

## Data Availability Statement

The original contributions presented in the study are included in the article/Supplementary Material, further inquiries can be directed to the corresponding authors.

## Author Contributions

QL and QM prepared the manuscript. MD and QL conducted the experiments and analyzed the data. BH designed the experiments. MG and PZ supervised the study. All authors have read and agreed to the published version of the manuscript.

## Conflict of Interest

The authors declare that the research was conducted in the absence of any commercial or financial relationships that could be construed as a potential conflict of interest.

## Publisher’s Note

All claims expressed in this article are solely those of the authors and do not necessarily represent those of their affiliated organizations, or those of the publisher, the editors and the reviewers. Any product that may be evaluated in this article, or claim that may be made by its manufacturer, is not guaranteed or endorsed by the publisher.

## Supplementary Material

Supplementary Figure 1 Southern blotting analysis of *MeNPF4.5* transgenic plants. WT, wild type; M, marker; OE22-OE34: *MeNPF4.5* overexpress transgenic plants. The probes were *HPT* in left three samples and *MeNPF4.5* in right three samples, and the restriction endinuclease was *Hind* III.
