# Supplementary figures and images for: MeNPF4.5 Improves Cassava Nitrogen Use Efficiency and Yield by Regulating Nitrogen Uptake and Allocation
Source: Front Plant Sci. 2022 Apr 25;13:866855. doi: 10.3389/fpls.2022.866855 (PMC9083203; doi:10.3389/fpls.2022.866855)

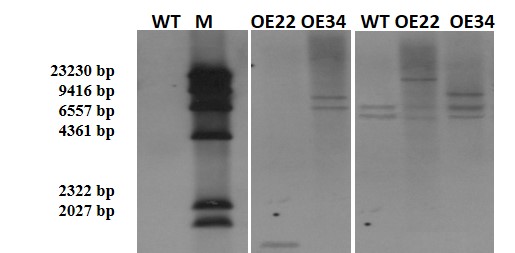

Supplement: Supplementary Figure 1 — Southern blotting analysis of MeNPF4.5 transgenic plants. WT, wild type; M, marker; OE22-OE34: MeNPF4.5 overexpress transgenic plants. The probes were HPT in left three samples and MeNPF4.5 in right three samples, and the restriction endinuclease was Hind III. [file Image_1.JPEG]
