# Supplementary material for: MeNPF4.5 Improves Cassava Nitrogen Use Efficiency and Yield by Regulating Nitrogen Uptake and Allocation
Source: Front Plant Sci. 2022 Apr 25;13:866855. doi: 10.3389/fpls.2022.866855 (PMC9083203; doi:10.3389/fpls.2022.866855)
Supplement: Supplementary file 2 [file Table_1.DOCX]

Supplementary table 1. The chemical properties of the tested soil

| pH | Organic matter (g kg^-1^) | Total N (g kg^-1^) | Total P  (g kg^-1^) | Total K (g kg^-1^) | Available N (mg kg^-1^) | Available P (mg kg^-1^) | Available K (mg kg^-1^) |
| --- | --- | --- | --- | --- | --- | --- | --- |
| 6.76 | 13.81 | 0.52 | 0.34 | 5.06 | 43.10 | 37.00 | 44.28 |

Note: N, nitrogen. K, potassium. P, phosphorus.
